# COMMUNITY ENGAGEMENT AND SERVICE LEARNING: PROMISING PEDAGOGICAL PRACTICES FOR TEACHING GERONTOLOGY

**DOI:** 10.1093/geroni/igz038.236

**Published:** 2019-11-08

**Authors:** Tina M Kruger

**Affiliations:** Indiana State University, Terre Haute, Indiana, United States

## Abstract

In terms of studying human development, gerontology is unique in that most college students have not experienced this aspect of the life course yet. While personal experience cannot be generalized, our students can at least relate to the idea of being a child, an adolescent, and a young adult. What they cannot do it relate to the experience of being old, and they may have limited contact with the older adult population, with the exception of grandparents, who tend to be viewed differently from older non-relatives. One way to facilitate students connecting with the older adult population is through community engagement or service-learning (CE/SL) projects. Such projects are ripe for facilitating learning, but there are also potential pitfalls to consider. Here we discuss the need for CE/SL in gerontology, theoretical and practical suggestions, and potential pitfalls to avoid.

